# The impact of bystanding to workplace bullying on symptoms of depression among women and men in industry in Sweden: an empirical and theoretical longitudinal study

**DOI:** 10.1007/s00420-012-0813-1

**Published:** 2012-09-02

**Authors:** R. Emdad, A. Alipour, J. Hagberg, I. B. Jensen

**Affiliations:** 1 Karolinska Institutet, Institute of Environmental Medicine, Division of Occupational and Enviromental Medicine, Unit of Intervention and Implementation Research, P.O. Box 210, 171 77 Stockholm, Sweden; 2 Department of Medical Sciences, Occupational and Environmental Medicine, Uppsala University, Uppsala, Sweden

**Keywords:** Job strain, Longitudinal, Industry, Bystanding workplace bullying, Depression, Model, Theory

## Abstract

**Background:**

Prospective studies on bystanding to workplace bullying and the health outcomes are scarce.

**Aim:**

To investigate the work environmental risk factors of depressive symptoms among bystanders to bullying in both women and men in four large industrial organizations in Sweden.

**Method:**

The number of respondents at four large industrial enterprises with more than one year at the workplace at T1: *n* = 2,563 (Women: *n* = 342; Men: *n* = 2,227). Bystanders to bullying at T1: *n* = 305 (Women: *n* = 30; Men: *n* = 275). The total number of those with symptoms of depression at T2: Women: *n* = 30; Men: *n* = 161. Two thousand one hundred and seventy-seven employees answered the questionnaire on T1 and T2 with an 18-month interval. “To have depressive symptoms” was defined as not having depressive symptoms at T1 but having depressive symptoms at T2.

**Results:**

The number of men who were bystanders to bullying was larger compared to women. However, the proportion of women who were bystanders to bullying and developed depressive symptoms 18 months later was higher in comparison with men (33.3 and 16.4 %, respectively). Further, “Being a bystander to bullying” 1.69 (1.13–2.53), “Rumors of changes in the workplace” 1.53 (1.10–2.14), “Reduced role clarity” 2.30 (1.21–4.32), “Lack of appreciation of being in the group” 1.76 (1.22–2.53) increased the risk of future symptoms of depression. “Job Strain” was not an adjusted risk factor for depression.

**Conclusion:**

Our results support previous findings that bystanding to workplace bullying is related to future depressive symptoms.

## Introduction

In the European Union, it is thought that one-third of the workforce experiences a mental health disorder in which depression is a significant factor (McDaid et al. 2005). Workplace bullying has been shown to cause symptoms of depression (Takaki et al. 2010), but there are only a few studies which have shown that bystanding to bullying behavior causes depression. However, evidence shows that workers who experience bullying in the workplace undergo a variety of negative psychological health outcomes such as depression (Nolfe et al. 2010; Raver and Nishii 2010; Fujishiro and Heaney 2009; Hammond et al. 2010; Roberts et al. 2004; Forman 2003; Mays et a. 1996; Agudelo-Suarez et al. 2009; Bhui et al. 2005; Kivimaki et al. 2003). In a study by Vingård et al. (2005), bullying was a risk indicator (Risk Ratio 1.5) for long-term sick-listing in women from the public sector in Sweden.

In a study by Vartia (2001), the effects of workplace bullying on the well-being and subjective stress of the targets and observers of bullying were investigated, with 85 % women, 15 % men. Both the targets of bullying and the witnesses reported more general stress and mental stress reactions than respondents from the workplaces with no bullying. In addition to negative target impact, this study emphasizes that even non-bullied witnesses report higher negativity and stress and, in contrast, indicate decreased work satisfaction and overall rating of their work experiences. This is in accordance with other studies exploring the impact of bullying on witnesses (Jennifer et al. 2003; Vartia 2001, 2003). Thus, bullying is not simply an interpersonal issue but is an organizational dynamic that impacts on all who are exposed—whether primarily or secondarily (Barling 1996).

The overwhelming feelings of stress can impact on not only the target of the bullying behavior, but also bystanders to the bullying. Workplace bullies, that is, people who belittle, humiliate, and threaten their co-workers, cost organizations billions of dollars each year (Georgakopoulos et al. 2011). In Sweden, depressive symptoms, clinical depression, anxiety, and distress are more common among women than among men (Bremberg 2006). These findings and other findings (Georgakopoulos et al. 2011) with regard to witnessing bullying are supported by Vartia (2001) and Mikkelsen and Einarsen (2001) who found similar results.

A Swedish national study carried out in three similar surveys in 1995, 1997, and 1999 estimated that an average of 8.6 % of men and 9.5 % of women reported being bullied in the last 12 months (Widmark et al. 2005). A strong association between workplace bullying and subsequent anxiety and depression, indicated by empirical research, suggests that bullying is an etiological factor for mental health problems (Brousse et al. 2008). Some bystanders might leave their jobs as a result of witnessing bullying (Rayner et al. 1999).

Barling’s discussion of primary and secondary victims of workplace violence suggests that secondary victims are employees who themselves were not victims but whose observations, fears, and expectations are changed as a result of being exposed to violence (Barling 1996). As such, bystanders to bullying could be considered as secondary targets, especially since bystanders report excessive workloads and role ambiguity (Jennifer et al. 2003). That is, in bullying work environments, bystanders most likely show symptoms of depression than non-exposed employees.

Twemlow et al. (2005) suggested that the bullying process is a triadic interaction enacted through the social roles of bully-victim-bystander. According to a number of investigations (Vartia 2001; Einarsen et al. 1998; O’Moore and Seigne 1998; Emdad et al. 2004), the perception of threat may lead to persistent emotional, psychosomatic, and psychiatric complications in victims. Investigators in this field of research have reached a similar conclusion (Einarsen 2000) that exposure to systematic and prolonged non-physical and non-sexual aggressive behaviors at work are highly damaging to the target’s health.

### Aim

The aim of the present longitudinal study was to investigate the work environmental risk factors of reported depressive symptoms among bystanders to bullying in both women and men in four large industrial organizations in Sweden.

## Subjects and method

### Study design and respondents

**Fig. 1:**
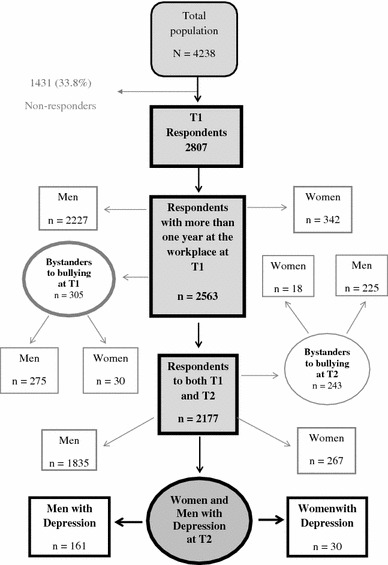
A schematic representation of participants in the study

This is a multicenter study entitled Work and Health in the Processing and Engineering Industries, the AHA Study (AHA is an abbreviation of the Swedish study title “Arbete och Hälsa inom process och verkstadsindustrin”). It was carried out at four large workplaces in Sweden during the years from 2000 to 2003. In this study, we will use the data collected in 2001 (T1) and 2003 (T2). Companies 1 and 2 are paper mills, company 3 is a steelworks, and company 4 is a truck manufacturer. The study was approved by the Ethical Committee of the Karolinska Institute (Dnr 00-012). The written informed consent of each of the employees was obtained. The population at the companies was mostly middle-aged and male-dominated (Bergstrom et al. 2008). Included in the present study were only those who had worked for at least 1 year at one of the four workplaces and who responded to the baseline questionnaire (T1: *n* = 2,563), and who were categorized as showing no symptoms of depression at T1 according to the HAD (see description of measures below), (Fig. 1).

### Screening

A comprehensive questionnaire addressing the employees’ health, lifestyle, and work-related factors was sent by mail to the entire workforce (from top management to the assembly line). This screening instrument was a compilation of valid questionnaires and was administered on two occasions (with an 18-month interval between assessments) during the course of the study.

### Measures

The objective of the AHA project is to develop a method of reinforcing and supporting sustainable health throughout one’s working life, achieving this through the implementation in companies and organizations of a method whereby measures aimed at promoting health and preventing ill health form a natural part of the work organization. The primary aim of the AHA method, which focuses on the psychosocial work environment, is to identify the factors in working life which can contribute to the health and well-being of the individual, work groups, and the organization. Surveying these factors provides valuable information about how the psychosocial work environment is perceived. The questionnaire used in the AHA method has been taken mainly from QPSNordic, which is an instrument for investigating psychosocial, social, and organizational conditions at the workplace. It has been developed and validated by a number of Nordic researchers and financed by the Nordic Council of Ministers (Lindström et al. 2000; Dallner et al. 2000).

*Job strain* (Theorell et al. 1998; Lindström et al. 2000; Dallner et al. 2000). The calculation of job strain was treated as suggested by the developers as follows: (1) Low strain, (2) Active, (3) Passive, and (4) High strain (Karasek 1979). In the analyses, we dichotomized strain as (1) High strain (2) No strain where 2 included Low Strain, Active, and Passive were combined.

In the present study, bystanders are referred to as co-workers who witnessed the bullying process.

The following questions were asked:

*Bystander to bullying* (Lindström et al. 2000; Dallner et al. 2000).

Have you noticed if anyone has been subjected to bullying/harassment at your workplace during the last 6 months?

(1) No (2) yes.

The median was calculated for the following items:

*Rumors of changes in the workplace* with regard to predictability of work (Lindström et al. 2000; Dallner et al. 2000).

(1) Very seldom or never (2) Seldom (3) Sometimes (4) Very often or always.

*Role Clarity* (Lindström et al. 2000; Dallner et al. 2000).

Are there clearly defined objectives for your work?

(1) Very seldom or never (2) Seldom (3) Sometimes (4) Very often or always.

Do you know which responsibilities you have?

(1) Very seldom or never (2) Seldom (3) Sometimes (4) Very often or always.

Do you know exactly what is required of you at work?

(1) Very seldom or never (2) Seldom (3) Sometimes (4) Very often or always.

*Appreciation of being in the group* (Lindström et al. 2000; Dallner et al. 2000).

(1) Very little or not at all (2) Little (3) Some (4) Pretty much (5) Very much.

The outcome variable depressive symptoms were assessed with the Hospital Anxiety and Depression Scale (HAD-depression). Response options were made on a 4-point Likert response scale (1: never; 2: sometimes, 3: often, 4: always). The scores were categorized into the three previously developed cut offs with <7: no sign of depression, 7–10 points: mild depression, 11 points and above: clinical depression. The categories were then dichotomized into <7 no depression and >7 depressed.

- I appreciate the same things as before.
- I can laugh and see things from the funny side.
- I am feeling lucky.
- I feel as if everything is moving slowly.
- I have lost interest in my appearance.
- I look forward to things with joy.
- I enjoy a good book or a good radio program or a good TV program.

### Statistical analysis

To analyze which variables would predict symptoms of depression at T2, we did the following: based on the review of the literature, a large set of relevant work environmental, individual, and demographic risk factors, in the questionnaire, was considered to be included in the Generalized Linear Model. The variables of age, gender, and bystanding to bullying, and job strain were forced to stay fixed in the model, even if they were statistically non-significant at the 5 % level. The main reason for choosing these variables was that these factors in the work environment have previously been shown to risk factors of depression. Variables with p-values not above 10 % level were re-entered in the model in later steps to see if they performed better when other variables were removed.

With regard to the question whether the respondent had been sexually harassed and whether the respondent had noticed if someone had been subjected to sexual harassment, the numbers were so few that we decided not to include them in the analysis.

## Results

Figure 1 shows a schematic representation of participants in the study. The total number of subjects in the four companies was *n* = 4,238. The total number of respondents with more than 1 year at the workplace at T1: *n* = 2,563 (Women: *n* = 342; Men: *n* = 2,227). Bystanders to bullying at T1, *n* = 305 (Women: *n* = 30; Men: *n* = 275). The total number of women with symptoms of depression at T2 was *n* = 30, and the total number of men with symptoms of depression at T2 was *n* = 161. The total number of employees who answered the questionnaire on both occasions (T1 and T2) was 2,177. Table 1 shows the frequencies of work-related, individual, and socio-demographic factors based only on the respondents who were included in the risk ratio model.

**Table 1:** Frequencies of socio-demographic, work-related, and individual factors for respondents at T1–T2 (*n* = 2,177)

Independent variables	Total^a^	New cases with depression (T2)	
		*n*	(%)
*Socio-demographic characteristics*			
Age categories			
Women			
19–43	114	14	12.3
44–65	153	16	10. 5
Men			
19–43	947	79	8.3
44–65	888	82	9.2
Education			
High School or lower education			
Women	233	28	12
Men	1,630	138	8.5
University			
Women	29	2	6.9
Men	189	23	12.2
*Work environmental characteristics*			
Bystander to bullying (yes)			
Women	18	6	33.3
Men	225	37	16.4
Bystander to bullying (no)			
Women	247	24	9.7
Men	1,590	120	7.3
High strain			
Yes	172	24	14
No	1,767	155	8.8
Rumors of changes in the workplace			
Yes	647	77	11.9
No	1,441	112	7.8
Role clarity			
Yes	1,966	175	8.9
No	69	14	20.3
*Individual characteristics*			
Appreciation of being in the group			
Yes	1,339	105	7.8
No	264	41	15.5

^a^Missing values are ignored

Although the total number of men who were bystanders to bullying was higher, the proportion of women who were bystanders to bullying and developed symptoms of depression 18 months later was higher compared to men (33.3 and 16.4 %, respectively). The table shows also that, among women, both age categories were overrepresented compared to men with regard to symptoms of depression. Table 1 also shows that men with higher education developed more symptoms of depression compared with women. Women with lower education developed more symptoms of depression.

Table 2 shows the risk ratio of symptoms of depression according to different levels of work environmental, individual, and socio-demographic characteristics, T1–T2, in the four large industrial enterprises in Sweden. The table shows that the relative risk of developing symptoms of depression which was significantly associated with “Being a bystander to bullying”, “Rumors of changes in the workplace”, “Role Clarity”, “Lack of appreciation of being in the group”, “Age”, “Gender” was not significantly associated with developing symptoms of depression. Job strain was not a significant risk factor for depression; although with regard to unadjusted model, it was significant.

**Table 2:** Adjusted and unadjusted risk ratios (RR) of depression according to socio-demographic, work environmental, and individual characteristics for respondents at T1–T2 in the four large industrial enterprises in Sweden (*n* = 2,177)

	Unadjusted RR	Adjusted RR (95 % CI)
*Socio-demographic characteristics*		
Age		
19–43	0.93 (0.70–1.22)	0.75 (0.54–1.04)
44–65		1
Gender		
Male	0.78 (0.54–1.13)	0.70 (0.42–1.03)
Female		1
*Work environmental*		
Bystander to bullying	2.26 (1.65–3.09)	1.69 (1.13–2.53)
Rumors of changes in the workplace	1.53 (1.16–2.02)	1.53 (1.10–2.14)
Reduced role clarity	2.28 (1.40–3.72)	2.30 (1.21–4.32)
Job strain		
High strain	1.59 (1.10–2.37) 1	1.34 (0.84–2.14) 1
*Individual characteristics*		
Lack of appreciation of being in the group	1.98 (1.42–2.78)	1.76 (1.22–2.53)

## Discussion

### The implications of main findings

The aim of the present study has been to explore whether bystanding to bullying, independent of other risk factors, explains symptoms of depression 18 months later in four large industrial organizations in Sweden. To the best of our knowledge, this is one of few studies to investigate development of symptoms of depression as a long-term effect of bystanding to workplace bullying. The results show, when adjusting for other factors of importance, the association between bystanding to bullying and the development of symptoms of depression remained significant. The risk of developing symptoms of depression within 1.5 years is increased by 1.69 (1.13–2.53). Different investigators suggest that bullying not only negatively affects the targets’ work production, but also adversely affects bystanders to bullying behavior (Jennifer et al. 2003; Vartia 2003). Bystanders more often leave their jobs as a result of their contact with bullying than do non-exposed workers (Rayner et al. 2002, p. 56; Vartia 2001).

Guilt is a widely accepted feature of depression (Ghatavi et al. 2002). In order to emphasize that bystanders to bullying are not a homogenous group, Emdad (2012, submitted article; 2012) has theoretically divided bystanders in four different subgroups according to their mentalization ability. According to Twemlow et al. (2005), when you mentalize about another human being, you put yourself in her shoes and try to understand your own inner impulses.

At the same time you try to understand and feel the other person’s feelings and thoughts. The first group has high mentalization ability; they can untangle and read the signals and can understand if anyone else suffers. This group of witnesses intervenes and tries to do something about the situation. “In some cases, bystanders choose not to get involved, which may lead to feelings of guilt. In other instances, they may try to help the target by finding ways to retaliate against the bully. In any case, the witnesses spend a great deal of time-discussing the bullying, resulting in potentially lower productivity for the organization” (Pearson and Porath 2005).

According to the model, group 2 has normal mentalization ability; they notice what is going on but are powerless over it. They do not tolerate bullying, but they do not dare to intervene (Lutgen-Sandvik and Tracy, *ibid*). They fear to lose their jobs. As a result, non-targeted co-workers also experience more stress, lower levels of job satisfaction, and higher turnover rates than individuals working in bully-free environments (Lutgen-Sandvik et al. 2007). Bystanders to bullying who develop symptoms of depression over time are in the subgroup number 2 in this theoretical model.

The third group in the model has low mentalization ability. They cannot see the health consequences of bullying. They tolerate bullying and ignore the processes that are going on. Group four has dysfunctional mentalization ability; they see bullying but blaming the victim. They do not participate, but believe that the victim has herself or himself to blame. Studies have shown that non-mentalizers quite often overestimate or underestimate aggression (Blair and Cipolotti 2000) and may therefore be surprised, for example, when somebody is frightened of them. “They tend to attribute negative intent to others when none is meant and are rigid and inflexible about their expectations of others. They are incapable of developing solutions to interpersonal problems that are acceptable to all parties; instead, solutions are biased in their favor (Twemlow et al. 2005).” Deficiency in mentalization stems from a relative deficiency of mentalizing in early attachment (Fonagy and Bateman 2006).

It was also shown (Table 2) that reduced role clarity was a predictor of depressive symptoms in the industrial settings. Worrall and Cooper (1998) and Lapido and Wilkinson (2002) reported reduced role clarity and increased work pressures as typical characteristics of organizational changes. Hence, negative acts associated with bullying in organizations characterized by change may primarily be related to task-oriented issues (Skogstad et al. 2007).

Reduced role clarity might provide a fertile ground for many bullies pick on a target that is competent in the group. They may target not only the vulnerable, but also those who threaten their sense of superiority or make them feel vulnerable (Yamada 2000, p. 4). “Lack of appreciation of being in the group” was a risk factor for developing symptoms of depression in this study. This finding is in line with Twemlow et al. (2005), Lutgen-Sandvik and McDermott (2008) who report that bullying behavior is much more complex than to be just a dyadic relationship between the bully and the victim of bullying. Thinking of bullying as a dyadic relationship, that is, involving only a bully and a target would lead to viewing it as just a subjective experience. As such, authorities may be less likely to believe target reports and take instantaneous corrective action.

One of the significant findings to emerge from this study is that “rumors of changes in the workplace”, further impact upon the employee’s mental health functioning. As shown in Table 1, although the total number of men who were bystanders to bullying was larger, the proportion of women who were bystanders to bullying and developed symptom of depression 18 months later was higher compared to men. This finding is in line with the results of a study by Skogstad et al. (2007). Their data from a sample of 2,408 Norwegian employees confirmed that different organizational changes were associated with task-related bullying at work and that exposure to more changes increased the likelihood of being bullied. Gender-based bullying has increased in the industrial settings as female workers have been employed in roles that were traditionally viewed as “male.” Despite this, there is little empirical evidence of the incidence of workplace bullying in the industry, gender-based or otherwise (van Barneveld and Jowett 2005).

In the present study, the respondents who did not appreciate, being in the group, showed signs of depression 18 months later. Workplace bullying in Sweden has often taken the form of bullying with a group of workers as the perpetrator, ‘ganging up’ on an isolated and vulnerable individual (Leymann 1996); (Zapf and Einarsen 2005). For example, the *Näringsdepartementet* (Ministry of Industry) paper states that a typical pattern of bullying can be identified in Sweden, which includes a spiral of mobbing behavior (Cited in Beale and Hoel 2010). The victim might experience fear, a sense of isolation, and insecurity at the prospect of meeting the bully in the group or visiting the location where the bullying has taken place or takes place; one is unable to attend meetings and may even vomit before, during or after the meeting, sometimes at the mere thought of the meeting. These are PTSD diagnostic criteria B4 and B5 (Kuehnel and LCSW 2010), and, in the long run, this approach-avoidance behavior could lead to clinical depression.

The results of the present study show that job strain was not a risk factor for depression. While control at work has generally been found to be related to high levels of satisfaction and low levels of experienced job stress (Hackman and Oldham 1980; Spector 1986), being exposed to workplace bullying should consequently by definition be characterized by gradually being deprived of control and possibilities to cope with bullying (Zapf and Einarsen 2005). In the present study, we would expect that the dimension of control in job strain would show a meaningful relationship with depression, but the results show that it is bystanding to bullying which is a risk factor for depression and not the job strain formulation.

### Methodological considerations

The majority of studies on workplace bullying are based on cross-sectional design. Podsakoff et al. (2003) suggested a temporal separation by introducing a time lag between the measurement of the predictor and criterion variables, in order to minimize the potential biasing effects of common methods variance. Thus, we used a design in which we collected data at two points in time separated by 18 months. The prospective design of our study did let us determine on the causal nature of the relationship between bystanding to workplace bullying and depression.

A previous study by Kivimaki et al. (2003) reported a strong association between workplace bullying and subsequent depression, suggesting that bullying is an etiological factor for mental health problems. In the present study, we decided to define depression as “not having depression at T1 but having depression at T2.” In this way, risk factors for depression, *inter alia*, bystanding to bullying could be better investigated. A reporting bias could be assumed as bystanding to workplace bullying, and depression was measured using self-reporting. This reporting bias is related to common method variance. One limitation was that the data on depression was based on self-reporting, which provides a range of depressive symptoms but not a depression diagnosis. Second, the bystanding to bullying question was very general, and different types of bullying were not specified. Third, our bullying data were pooled from self-reporting. Validated instruments were used to measure depressive symptoms (HAD-scale). One limitation of the study was the very low number of women in the study and the still lower number of cases among women.

### Recommendations

Our data suggests that frequent bystanding to bullying may be a warning sign for developing future symptoms of depression. Our study gives grounds for actively collecting information on bullying behavior as part of screening during health control in occupational health services. Moreover, bullying should be the focus of preventive work in the industry. In conclusion, the results support the notion that bullying is not only a dyadic target-bully issue to be resolved. It has to be seen as a triadic relationship between bully, victim, and bystander and as a structural, organizational problem where many bystanders as well as targets suffer and are at risk of future health problems. Bystanders and the whole organization are involved in the process of bullying behavior, and, in turn, intervention programs should be focused on the whole workplace system.
